# Supplementary material for: MetaRibo-Seq measures translation in microbiomes
Source: Nat Commun. 2020 Jun 29;11:3268. doi: 10.1038/s41467-020-17081-z (PMC7324362; doi:10.1038/s41467-020-17081-z)
Supplement: Supplementary file 10 — Supplementary Data 7 [file 41467_2020_17081_MOESM10_ESM.zip › File2/Confidence_VeryHigh_Taxonomy/279310_out.krona.html]

Javascript must be enabled to view this page.

members
magnitude
magnitudeUnassigned
count
unassigned
taxon
rank

279310\_out

12

7
superkingdom
2

1239
7
phylum

class
7
186801

order
7
186802

31979
5
family

genus
1485
2
5

SRS021948\_contig\_number\_contig-100\_30972.30972SRS043667\_contig\_number\_contig-100\_8636.8636


SRS015264\_contig\_number\_contig-100\_3379.87555SRS148253\_contig\_number\_contig-100\_481.482
2293024
2
species


SRS014287\_contig\_number\_contig-100\_9880.86488
2293029
1
species

186804
family
1

1257
genus
1

1
species

SRS011306\_contig\_number\_contig-100\_334.218654
1261

186803
1
family

1506553
genus
1

1
species

SRS045244\_contig\_number\_contig-100\_28635.28636
208479

5

SRS016335\_contig\_number\_contig-100\_22790.57987SRS043841\_contig\_number\_contig-100\_28009.28010SRS075984\_contig\_number\_24924SRS142890\_contig\_number\_contig-100\_2950.163598SRS971276\_contig\_number\_9107
